# Antidepressant-Like Effect of the Leaves of *Pseudospondias microcarpa* in Mice: Evidence for the Involvement of the Serotoninergic System, NMDA Receptor Complex, and Nitric Oxide Pathway

**DOI:** 10.1155/2015/397943

**Published:** 2015-10-11

**Authors:** Donatus Wewura Adongo, Kennedy Kwami Edem Kukuia, Priscilla Kolibea Mante, Elvis Ofori Ameyaw, Eric Woode

**Affiliations:** ^1^Department of Pharmacology, Faculty of Pharmacy & Pharmaceutical Sciences, College of Health Sciences, Kwame Nkrumah University of Science & Technology, Kumasi, Ghana; ^2^Department of Pharmacology, Medical School, University of Ghana, Accra, Ghana; ^3^Department of Biomedical and Forensic Sciences, School of Biological Science, University of Cape Coast, Cape Coast, Ghana

## Abstract

Depression continues to be a major global health problem. Although antidepressants are used for its treatment, efficacy is often inconsistent. Thus, the search for alternative therapeutic medicines for its treatment is still important. In this study, the antidepressant-like effect of *Pseudospondias microcarpa* extract (30–300 mg kg^−1^, *p.o.*) was investigated in two predictive models of depression—forced swimming test and tail suspension test in mice. Additionally, the mechanism(s) of action involved were assessed. Acute treatment with the extract dose dependently reduced immobility of mice in both models. The antidepressant-like effect of the extract (100 mg kg^−1^, *p.o.*) was blocked by *p*-chlorophenylalanine and cyproheptadine but not prazosin, propranolol, or yohimbine. Concomitant administration of d-cycloserine and the extract potentiated the anti-immobility effect. In contrast, d-serine, a full agonist of glycine/NMDA receptors, abolished the effects. Anti-immobility effects of PME were prevented by pretreatment of mice with L-arginine (750 mg kg^−1^, i.p.) and sildenafil (5 mg kg^−1^, i.p.). On the contrary, pretreatment of mice with L-NAME (30 mg kg^−1^, i.p.) or methylene blue (10 mg kg^−1^, i.p.) potentiated its effects. The extract produces an antidepressant-like effect in the FST and TST that is dependent on the serotoninergic system, NMDA receptor complex, and the nitric oxide pathway.

## 1. Introduction

Major depression is one of the most common psychiatric disorders and is characterized by change in mood and lack of interest in the surroundings as well as psychosocial and physical impairment [1, 2]. Depression is an important global public health issue, because of the relatively high lifetime prevalence ranging from 2% to 15% and also because it is associated with substantial disability [3]. The World Health Organization estimates that, by 2020, major depression will become the second largest cause of global disease problems in the world, only behind ischemic heart disease [4, 5].

There are several antidepressant drugs available, most of them affecting directly or indirectly the monoaminergic system [6]. These classical antidepressant agents are designed to increase monoamine transmission, either by inhibiting neuronal reuptake (imipramine, fluoxetine, or desipramine) or by inhibiting degradation (iproniazid) [7, 8]. However, a major limitation of these antidepressants is side effects such as sedation, blurred vision, constipation, seizures, sexual dysfunction, and weight gain [3, 9]. Furthermore, although these are effective in treating most depressive episodes, a significant proportion of depressed patients do not display signs of mood improvement until 2-3 weeks after the start of the treatment [10]. Accordingly, natural plants may be important sources of new antidepressant drugs and the safety of such plant extracts may be better than that of synthetic antidepressants [11, 12]. It is therefore desirable to research and develop more effective antidepressants with fewer adverse effects. Plant extracts are some of the most attractive sources of new drugs and have been shown to produce promising results for the treatment of depression. For instance, the excellent patient acceptance of St. John's Wort (*Hypericum perforatum* L., Hypericaceae) and its extensive use in Europe and USA for the treatment of mood disorders, especially conditions of mild to moderate depression, has drawn attention to plant extracts as potential sources of highly desirable, new, and innovative antidepressant agents [13, 14].* Pseudospondias microcarpa* is also one of such plants used for managing various diseases including CNS disorders [15]. However, despite the wide use of the plant, there is no data in literature on its probable antidepressant activity.

Therefore, the present study evaluated the antidepressant-like effect of the hydroethanolic extract obtained from the leaves of* Pseudospondias microcarpa* (PME) in two predictive models of depression: forced swimming test (FST) and tail suspension test (TST) in mice. Moreover, the mechanisms through which PME elicits its antidepressant-like action were investigated in the TST.

## 2. Materials and Methods

### 2.1. Collection of Plant Material

Fresh leaves of* Pseudospondias microcarpa* were collected from the campus of Kwame Nkrumah University of Science and Technology (KNUST), Kumasi, near the Department of Agricultural Engineering (6°40.626′N, 1°34.041′W), during the month of August 2010, and authenticated at the Department of Herbal Medicine, Faculty of Pharmacy and Pharmaceutical Sciences, College of Health Sciences, KNUST, Kumasi, Ghana. A voucher specimen (KNUST/HM1/2013/L005) was kept at the herbarium of the faculty.

### 2.2. Plant Extraction

Leaves of the plant were room-dried for seven days and pulverized into fine powder. The powder was extracted by cold percolation with 70% (v/v) ethanol in water over a period of 72 h and the resulting extract concentrated into a syrupy mass under reduced pressure at 60°C in a rotary evaporator. It was further dried in a hot air oven at 50°C for a week and kept in a refrigerator for use. The yield was 20.5% (w/w). In this study, the crude extract is subsequently referred to as PME or extract.

### 2.3. Animals

Male ICR mice (20–25 g) were purchased from the Noguchi Memorial Institute for Medical Research, Accra, Ghana, and kept in the animal house of the Department of Pharmacology, Kwame Nkrumah University of Science and Technology, Kumasi, Ghana. The animals were housed in groups of 5 in stainless steel cages (34 cm × 47 cm × 18 cm) with soft wood shavings as bedding and housing conditions controlled—temperature maintained at 24-25°C, relative humidity 60–70%, and 12 h light-dark cycle. They had free access to tap water and food (commercial pellet diet, GAFCO, Tema, Ghana). A period of at least one week for adaptation to the laboratory facilities was allowed. The studies were conducted in accordance with accepted principles for laboratory animal use and care (NRC, 2010). Approval for this study was obtained from the faculty Ethics Committee.

### 2.4. Drugs and Chemicals

Desipramine hydrochloride, para-chlorophenylalanine (pCPA), *α*-methyl-para-tyrosine methyl ester (AMPT), d-cycloserine (DCS, D-4-amino-3-isoxazolidine), d-serine (DS), 5-hydroxy-L-tryptophan (5-HTP), N-nitro-L-arginine methyl ester (L-NAME), L-arginine, norepinephrine, yohimbine, and methylene blue were purchased from Sigma-Aldrich Inc., St. Louis, MO, USA. Cyproheptadine was purchased from LETAP Pharmaceuticals Ltd., Accra, Ghana. Sildenafil and prazosin hydrochloride were from Pfizer, USA. Propranolol hydrochloride was from Watson Pharma Private Ltd., India, and fluoxetine hydrochloride was from Prozac, Eli Lilly and Company Ltd., Basingstoke, England.

### 2.5. Forced Swimming Test

This experiment was performed according to the procedure of Porsolt et al. [16] with modifications. Briefly, mice were pretreated with vehicle (10 mL kg^−1^ of 0.9% NaCl, i.p.), PME (30, 100 and 300 mg kg^−1^,* p.o.*), fluoxetine (3, 10, and 30 mg kg^−1^,* p.o.*), or desipramine (3, 10, and 30 mg kg^−1^, i.p.) 60 min (*p.o.*) or 30 min (i.p.) before being placed individually in polypropylene cylinders (height 25 cm, diameter 10 cm) containing 10 cm of water, maintained at 25°C. With a public domain software JWatcher, version 1.0 (University of California, Los Angeles, USA, and Macquarie University, Sydney, Australia), behavioural assessment was measured during the last 4 min of the 6-minute test period according to Detke et al. [17]. Three different behaviours were rated: (1) immobility: mice were judged to be immobile when they remained floating passively in the water, (2) swimming: mice were judged to be swimming if they were making active swimming motions, more than necessary, to solely maintain their head above water, and (3) climbing: mice were judged to be climbing when they were making active movements in and out of the water with their forepaws, usually directed against the walls. Duration of immobility, swimming, and climbing was measured.

### 2.6. Tail Suspension Test

The tail suspension test (TST) was conducted as initially described by Steru et al. [18] with modifications [19, 20]. Animals were similarly grouped as in the FST. One hour after oral administration and 30 min after intraperitoneal injection of test compounds, mice were individually suspended by the tail from a horizontal ring-stand bar raised 30 cm above the floor using adhesive tape placed 1 cm from the tip of tail and positioned such that the base of their tail was aligned with the horizontal plane. Test sessions lasted for 6 min and were videotaped. Behaviours for the last 4 min of the 6-minute period were then analysed. Behaviours rated were as follows: (1) immobility: a mouse was judged to be immobile when it hung by its tail without engaging in any active behaviour, (2) swinging: a mouse was judged to be swinging when it continuously moved its paws in the vertical position while keeping its body straight and/or it moved its body from side to side, (3) curling: a mouse was judged to be curling when it engaged in active twisting movements of the entire body, and (4) pedalling: it is defined as when the animal moved its paws continuously without moving its body.

### 2.7. Mechanism(s) of Action

The TST presents some advantages over the FST in allowing an objective measure of immobility and does not induce hypothermia by immersion in water [21, 22]. Thus, this model was therefore used to assess the possible mechanism of action.

#### 2.7.1. Serotoninergic Depletion

In order to investigate the possible contribution of the serotoninergic system on the effect of PME in the TST, mice were pretreated with para-chlorophenylalanine (pCPA). pCPA is known to reduce the concentration of brain serotonin by inhibiting its biosynthesis [23]. In the present experiment, mice were injected i.p. either with saline (control group) or with pCPA. pCPA was administered at the dose of 300 mg kg^−1^ once daily for 3 consecutive days. On the fourth day (24 h after the last pCPA administration), mice received PME (100 mg kg^−1^,* p.o.*), FLX (10 mg kg^−1^,* p.o.*), DES (10 mg kg^−1^, i.p.), or saline 60 min (*p.o.*) or 30 min (i.p.) before the test.

#### 2.7.2. 5-HTP-Induced Head-Twitch Response

PME (100 mg kg^−1^,* p.o.*), FLX (10 mg kg^−1^,* p.o.*), DES (10 mg kg^−1^, i.p.), or saline were administered 60 min (*p.o.*) or 30 min (i.p.) before intraperitoneal administration of 5-HTP (200 mg kg^−1^). Mice were then placed into plastic cages and the number of head-twitches (rapid movements of the head with little or no involvement of the trunk) was counted for 8 min (from 15 to 23 min) after the injection of 5-HTP [24, 25].

#### 2.7.3. Effects of Some Antagonists on PME Actions in the TST

Appropriate doses for antagonists were selected from literature [26] as well as pilot experiments, and doses that do not modify immobility were used. Groups of mice received saline or antagonists (cyproheptadine, 8 mg kg^−1^,* p.o.*, a 5-HT_2_ receptor antagonist; prazosin, 3 mg kg^−1^,* p.o.*, a selective *α*_1_-receptor antagonist; propranolol, 3 mg kg^−1^,* p.o.*, *β*-receptor antagonist; or yohimbine 3 mg kg^−1^,* p.o.*, *α*_2_-receptor antagonist) 30 min before vehicle or PME (100 mg kg^−1^,* p.o.*) and were assessed 45 min later for immobility time in the TST.

#### 2.7.4. Potentiation of Norepinephrine Toxicity

This was done to assess the possible involvement of the noradrenergic system in the antidepressant-like effect of PME. Mice were randomly assigned to test groups of 10 subjects. Mice were pretreated with vehicle (10 mL kg^−1^ of 0.9% NaCl, i.p.), PME (30, 100, and 300 mg kg^−1^,* p.o.*), fluoxetine (30 mg kg^−1^,* p.o.*), or desipramine (30 mg kg^−1^, i.p.) 60 min (*p.o.*) or 30 min (i.p.) prior to the s.c. injection of the sublethal dose of noradrenaline (3 mg kg^−1^). Mice were then placed in plastic cages with free access to food and water, and mortality rate was assessed 48 hours after dosing.

#### 2.7.5. N-Methyl-d-aspartate (NMDA) Interaction

To evaluate the effects of d-cycloserine (DCS) and d-serine (DS) in the mouse TST, the drugs were administered 30 min before the test to different experimental groups of animals. Immobility time was compared with a control group in which saline was injected 30 min before the test. For the present report, the doses of these drugs were chosen based on a pilot study and in accordance with previous studies [10, 27].

To evaluate the possible involvement of the activation of the NMDA receptor system in the antidepressant effect of PME in the TST, subeffective doses of d-cycloserine (2.5 mg kg^−1^, i.p.) and d-serine (600 mg kg^−1^, i.p.) were separately administered 15 min before administration of PME (100 mg kg^−1^,* p.o.*), FLX (10 mg kg^−1^,* p.o.*), DES (10 mg kg^−1^, i.p.), or saline. Forty-five minutes after administration, the mice were assessed in the TST for duration of immobility.

#### 2.7.6. Involvement of L-Arginine-NO-cGMP Pathway

An appreciable number of studies have attributed a significant role to the L-arginine-NO-cGMP pathway in the pathophysiology of depression [28, 29]. Therefore, the possible participation of this pathway in the antidepressant effect of PME was investigated. Mice were pretreated with a subeffective dose of L-arginine [750 mg kg^−1^, i.p., a precursor of nitric oxide (NO)] or vehicle 15 min before PME (100 mg kg^−1^,* p.o.*) administration and assessed 45 min later for immobility time. In separate experiments, the enhanced anti-immobility effect of PME with L-NAME [30 mg kg^−1^, i.p., a nonselective nitric oxide synthase (NOS) inhibitor] or methylene blue [10 mg kg^−1^, i.p., an inhibitor of nitric oxide synthase and an inhibitor of soluble guanylate cyclase (sGC)] was investigated. Mice were administered with these inhibitors 15 min before PME or vehicle and assessed 45 min later for immobility time in the TST.

To observe the role of cyclic guanosine monophosphate (cGMP) in the antidepressant action of PME, mice received an injection of sildenafil [5 mg kg^−1^, i.p., a phosphodiesterase 5 inhibitor (PDE5)] or vehicle 15 min before PME (100 mg kg^−1^,* p.o.*). Forty-five minutes after PME administration, the mice were subjected to TST to evaluate immobility duration.

### 2.8. Rotarod Test

In FST and TST, false-positive results can be obtained with certain drugs, in particular, psychomotor stimulants, which decrease immobility time by stimulating locomotor activity [30, 31]. Therefore, the effect of PME on motor coordination was assessed using a rotarod apparatus (Ugo Basile, model 7600, Cormerio, Milan, Italy). The rotarod consisted of a rotating rod (diameter of 3 cm) and individual compartments for each mouse. Mice were trained for 3 days before the test to stay on the rotating rod (speed 20 rpm) for at least 5 min. On the test day, mice were randomly divided into seven groups: saline-treated control group, diazepam group (0.1, 0.3, and 1 mg kg^−1^, i.p.), and PME group (30, 100, and 300 mg kg^−1^,* p.o.*). One hour (*p.o.*) or 30 (i.p.) min after administration of test compounds, mice were put on the rotating rod and latency until fall during the 5 min session was recorded. Animals that stayed on the bar for more than 5 min were given the maximum score of 5 min.

### 2.9. Statistical Analysis

In all experiments, a sample size of 5–10 animals was utilized. All data are presented as mean ± SEM. To compare differences between groups, one-way ANOVA was performed with Newman-Keuls test as* post hoc*. In some instances, behavioural data were analysed using two-way ANOVA followed by Bonferroni's test as* post hoc*. GraphPad Prism for Windows 5 (GraphPad Software, San Diego, CA, USA) was used for all statistical analysis. *P* < 0.05 (Newman-Keuls test or Bonferroni's test) was considered statistically significant.

## 3. Results

### 3.1. Forced Swimming Test

**Figure 1:**
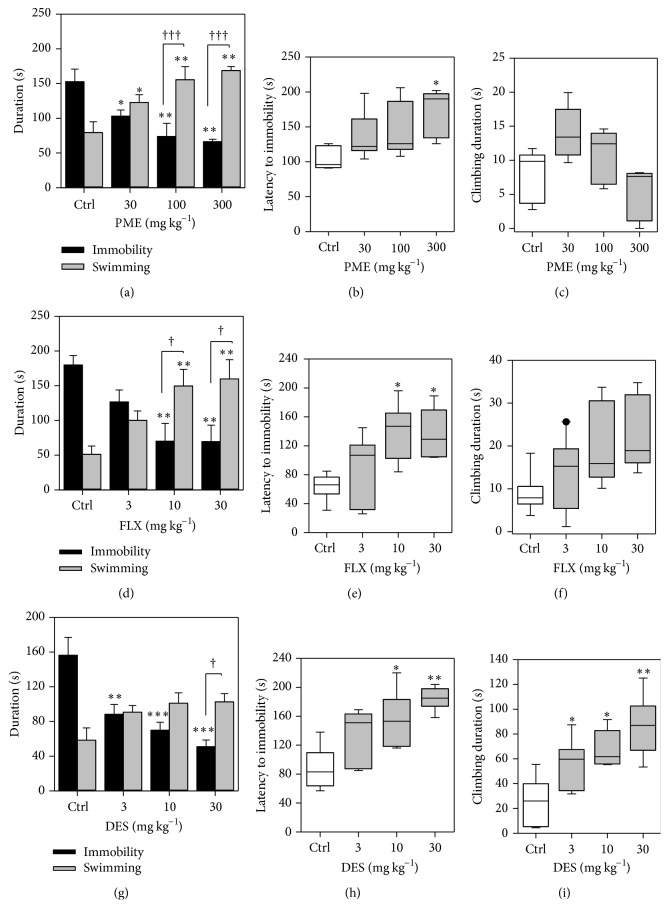
Performance of mice in the FST: behavioural assessment including immobility and swimming duration (a, d, and g), immobility latency (b, e, and h), and climbing duration (c, f, and i) after acute treatment of mice with PME, fluoxetine, and desipramine. PME (30–300 mg kg^−1^) and FLX (3–30 mg kg^−1^) were* p.o.* administered 60 min before behavioural assessment. DES (3–30 mg kg^−1^) was i.p. injected 30 min before the test. Data are expressed as group mean ± SEM (*n* = 5). The lower and upper margins of the boxes represent the 25th and 75th percentiles, with the extended arms representing the 10th and 90th percentiles, respectively. The median is shown as the horizontal line within the box and symbols represent outliers. Significantly different from control: ^*∗*^ *P* < 0.05; ^*∗∗*^ *P* < 0.01; ^*∗∗∗*^ *P* < 0.001 (one-way ANOVA followed by Newman-Keuls* post hoc* test) and significant difference when immobility and swimming were compared to each other. ^†^ *P* < 0.05; ^†††^ *P* < 0.001 (two-way repeated measures ANOVA followed by Bonferroni's* post hoc*).

Figure 1 depicts the effect of acute administration of PME (30–300 mg kg^−1^,* p.o.*) and the classical antidepressant drugs fluoxetine (3–30 mg kg^−1^,* p.o.*) and desipramine (3–30 mg kg^−1^, i.p.) on mice behaviours in the FST.

In this test, ANOVA revealed that all doses of PME significantly decreased the immobility time (*F*_3,16_ = 7.995, *P* = 0.0018) and increased swimming time (*F*_3,16_ = 8.462, *P* = 0.0013) of mice in the FST by a maximum of 62.83 ± 9.98% and 94.53 ± 17.31%, respectively (Figure 1(a)). ANOVA revealed that PME did not significantly affect latency to immobility (*F*_3,16_ = 3.062, *P* = 0.0583). However,* post hoc* analysis showed statistical significance at 300 mg kg^−1^ (*P* < 0.05). Climbing time was not affected. In Figure 1(d), ANOVA analysis revealed that fluoxetine significantly decreased the immobility time (*F*_3,16_ = 7.995, *P* = 0.0043) and increased swimming time (*F*_3,16_ = 8.462, *P* = 0.0060) of mice in FST reaching statistical significance at 10 and 30 mg kg^−1^ (both *P* < 0.01). Latency to immobility (*F*_3,16_ = 4.490, *P* = 0.0181) was significantly affected but not climbing behaviour. In Figure 1(e), swimming behaviour of desipramine was not significantly affected (*F*_3,16_ = 3.350, *P* = 0.669). However, ANOVA revealed a significant reduction of immobility time (*F*_3,16_ = 11.95, *P* = 0.0002) and significant increase in immobility latency (*F*_3,16_ = 6.785, *P* = 0.0037) (Figure 1(e)). Climbing time was also significantly increased (*F*_3,16_ = 6.850, *P* = 0.0035).

### 3.2. Tail Suspension Test

**Figure 2:**
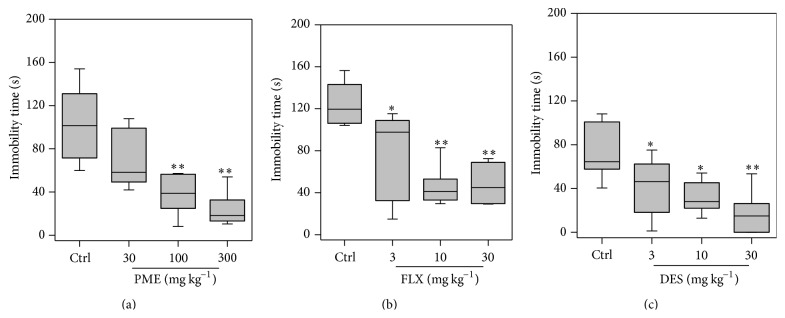
Effects of PME (30–300 mg kg^−1^), fluoxetine (3–30 mg kg^−1^), and desipramine (3–30 mg kg^−1^) on the total duration of immobility in the TST. The values represent mean ± SEM (*n* = 5). The lower and upper margins of the boxes represent the 25th and 75th percentiles, with the extended arms representing the 10th and 90th percentiles, respectively. The median is shown as the horizontal line within the box. Significantly different from control: ^*∗*^ *P* < 0.05; ^*∗∗*^ *P* < 0.01 (one-way ANOVA followed by Newman-Keuls* post hoc* test).

**Figure 3:**
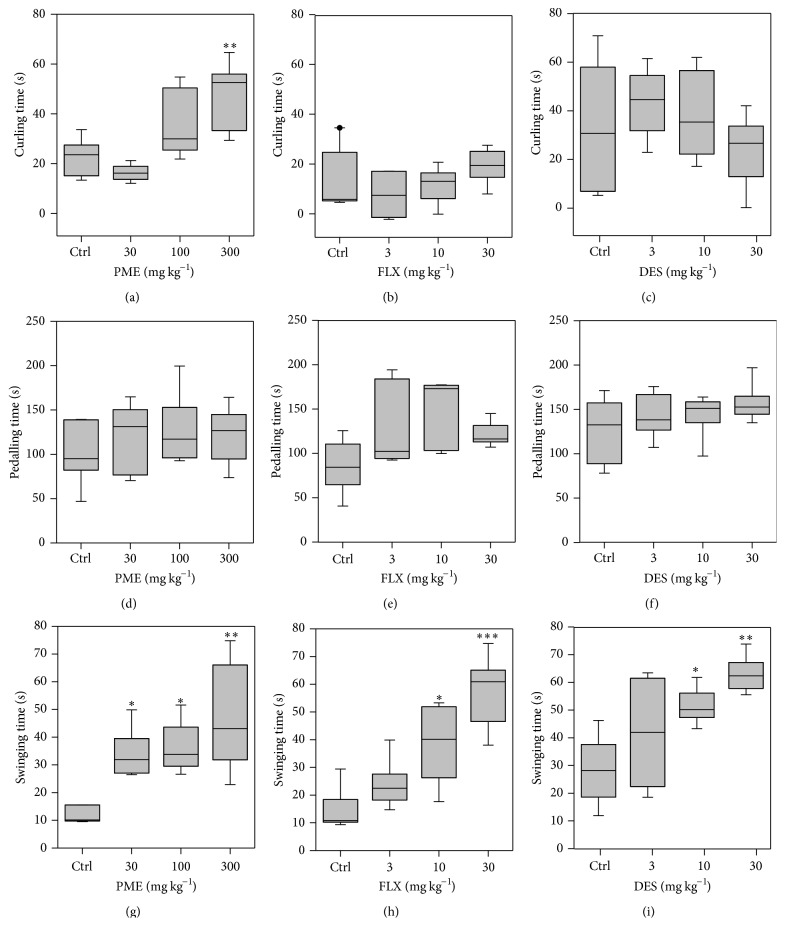
Performance of mice in the TST: behavioural assessment including curling (a, b, and c), pedaling (e, f, and g), and swinging (g, h, and i) after acute treatment of mice with PME, fluoxetine, and desipramine. PME (30–300 mg kg^−1^) and FLX (3–30 mg kg^−1^) were* p.o.* administered 60 min before behavioural assessment. DES (3–30 mg kg^−1^) was i.p. injected before the test. Data are expressed as group mean ± SEM (*n* = 5). The lower and upper margins of the boxes represent the 25th and 75th percentiles, with the extended arms representing the 10th and 90th percentiles, respectively. The median is shown as the horizontal line within the box. Significantly different from control: ^*∗*^ *P* < 0.05; ^*∗∗*^ *P* < 0.01; ^*∗∗∗*^ *P* < 0.001 (one-way ANOVA followed by Newman-Keuls* post hoc* test).

Figures 2 and 3 represent the effect of acute administration of PME (30–300 mg kg^−1^,* p.o.*) and the classical antidepressant drugs fluoxetine (3–30 mg kg^−1^,* p.o.*) and desipramine (3–30 mg kg^−1^, i.p.) on mice behaviours in the TST. Administration of PME (30–300 mg kg^−1^,* p.o.*) 1 h before the test period significantly decreased the immobility periods of mice by maximum of 80.98 ± 18.75% when compared to control group, indicating significant antidepressant-like activity. Newman-Keuls* post hoc* test indicated statistically significant anti-immobility effects of PME at doses of 100–300 mg kg^−1^ (*P* < 0.01 at 100 and 300 mg kg^−1^). Results in Figure 3 show that PME did not significantly affect pedalling but caused an increase in time spent swinging (*F*_3,16_ = 6.951, *P* = 0.0033) and curling (*F*_3,16_ = 7.580, *P* = 0.0022). Fluoxetine significantly increased anti-immobility effects by a maximum of 62.21 ± 25.07%. Swinging time was also significantly increased (*F*_3,16_ = 11.59, *P* = 0.0003) reaching statistical significance at 10 mg kg^−1^ (*P* < 0.05) and 30 mg kg^−1^ (*P* < 0.01). However, ANOVA did not indicate any significant effect of fluoxetine on pedaling (*F*_3,16_ = 2.039, *P* > 0.05) or curling (*F*_3,16_ = 1.246, *P* = 0.326) times. Administration of desipramine significantly reduced immobility time in a dose dependent manner by a maximum of 80.10 ± 17.38%. Swinging time was also significantly increased (*F*_3,16_ = 6.248, *P* = 0.0052). Just like fluoxetine, ANOVA did not reveal any significant effect of desipramine on pedaling and curling times.

### 3.3. Mechanism(s) of Antidepressant Action of PME

#### 3.3.1. Pretreatment with pCPA

**Figure 4:**
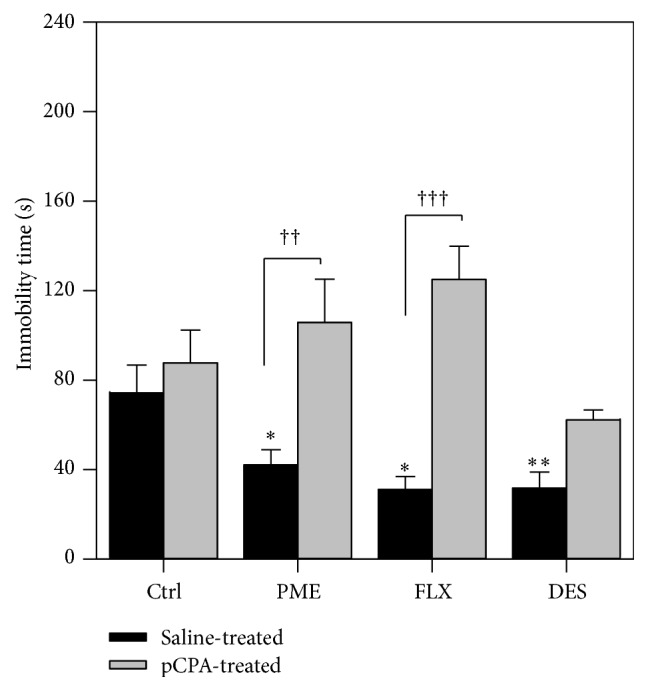
Effects of pCPA (300 mg kg^−1^, i.p. for 3 consecutive days) pretreatment on the behavioural response of PME (100 mg kg^−1^,* p.o.*), fluoxetine (10 mg kg^−1^,* p.o.*), and desipramine (10 mg kg^−1^, i.p.) in the tail suspension test. Data are presented as group mean ± SEM (*n* = 5). ^*∗*^ *P* < 0.05; ^*∗∗*^ *P* < 0.01 versus vehicle-treated animals (one-way ANOVA followed by Newman-Keuls' test). Significant difference between treatments: ^††^ *P* < 0.01; ^†††^ *P* < 0.001 (two-way ANOVA followed by Bonferroni's test).

The results in Figure 4 show that pCPA alone (300 mg kg^−1^ for 3 consecutive days) did not modify the immobility time, while pretreatment of mice with pCPA significantly blocked the reduction in the immobility time elicited by PME (100 mg kg^−1^,* p.o.*) in the TST (*F*_1,24_ = 40.45, *P* = 0.0002). Fluoxetine, the selective serotonin reuptake inhibitor (SSRI), reduced immobility in saline-pretreated mice. However, it significantly increased immobility in pCPA-pretreated animals when compared with the corresponding group given saline. Prior administration of pCPA did not alter the response of desipramine.

#### 3.3.2. 5-HTP-Induced Head-Twitch Response in Mice

**Figure 5:**
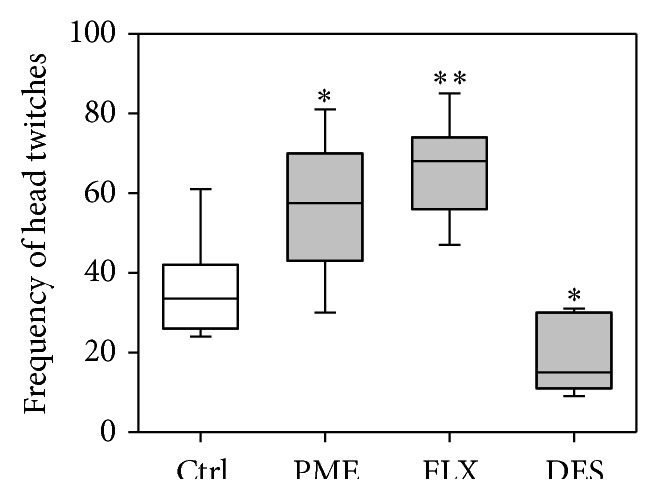
Effects of PME (100 mg kg^−1^), FLX (10 mg kg^−1^), and DES (10 mg kg^−1^) on the number of 5-HTP-induced head twitches in mice. Data are expressed as mean ± SEM (*n* = 5). The lower and upper margins of the boxes represent the 25th and 75th percentiles, with the extended arms representing the 10th and 90th percentiles, respectively. The median is shown as the horizontal line within the box. ^*∗*^ *P* < 0.05; ^*∗∗*^ *P* < 0.01 compared with the saline-treated control (one-way ANOVA followed by Newman-Keuls* post hoc* test).

As shown in Figure 5, PME and fluoxetine potentiated the number of head-twitch responses by maximum of 54.08% and 80.88% as compared to the control, respectively. Unlike PME and fluoxetine, desipramine significantly decreased the number of head-twitch responses in comparison to the control group (*P* < 0.05).

#### 3.3.3. Effects of Antagonists on PME Actions in the TST

**Figure 6:**
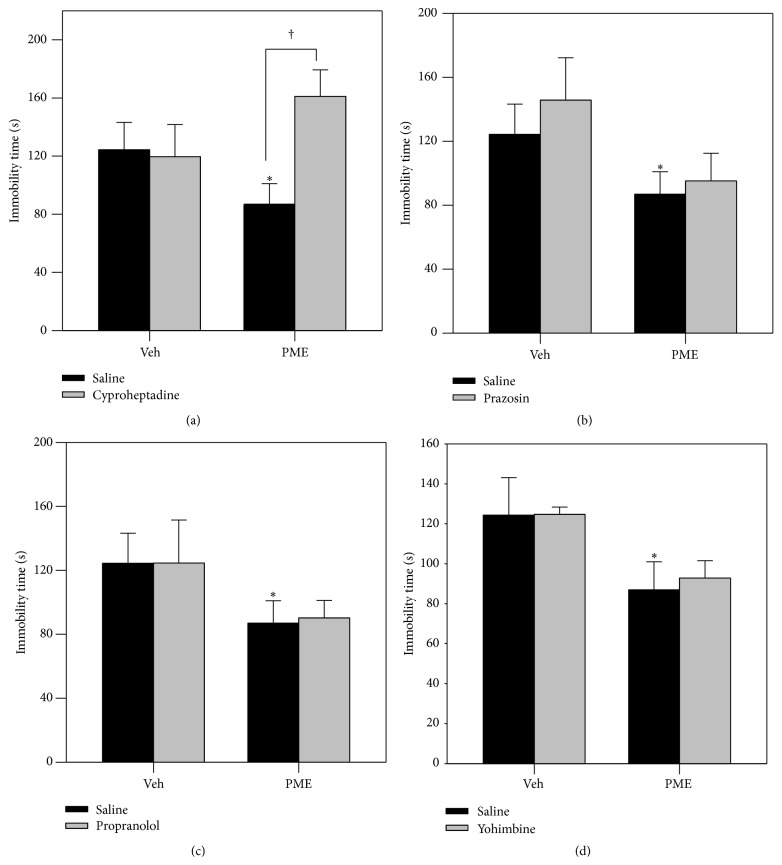
Effect of pretreatment of mice with cyproheptadine (8 mg kg^−1^,* p.o.*, a 5-HT_2_ receptor antagonist, panel (a)), prazosin (3 mg kg^−1^,* p.o.*, a selective *α*_1_-receptor antagonist, panel (b)), propranolol (3 mg kg^−1^,* p.o.*, *β*-receptor antagonist, panel (c)), and yohimbine (3 mg kg^−1^,* p.o.*, *α*_2_-receptor antagonist, panel (d)) on PME- (100 mg kg^−1^,* p.o.*) induced reduction in immobility time in the TST. Each column represents the mean ± SEM (*n* = 6). ^*∗*^ *P* < 0.05 as compared with the vehicle-treated control. ^†^ *P* < 0.05 as compared with the group pretreated with vehicle and PME.

Cyproheptadine (8 mg kg^−1^,* p.o.*), prazosin (3 mg kg^−1^,* p.o.*), propranolol (3 mg kg^−1^,* p.o.*), and yohimbine (3 mg kg^−1^,* p.o.*) were administered 30 min before PME and the tail suspension test was performed 45 min after PME administration. The anti-immobility effect caused by PME (100 mg kg^−1^,* p.o.*) was significantly prevented by pretreatment of mice with cyproheptadine (Figure 6(a)). Prazosin (Figure 6(b)), propranolol (Figure 6(c)), and yohimbine (Figure 6(d)) had no effect on the anti-immobility effect of the extract.

#### 3.3.4. Potentiation of Norepinephrine Toxicity

As shown in Table 1, injection of the sublethal dose of noradrenaline (3 mg kg^−1^, s.c.) caused no mortality in PME and FLX treated mice. However, desipramine pretreatment potentiated markedly and significantly NE toxicity in mice.

#### 3.3.5. Effect of Joint Administration of d-Serine or DCS and PME, FLX, or DES in the TST

**Figure 7:**
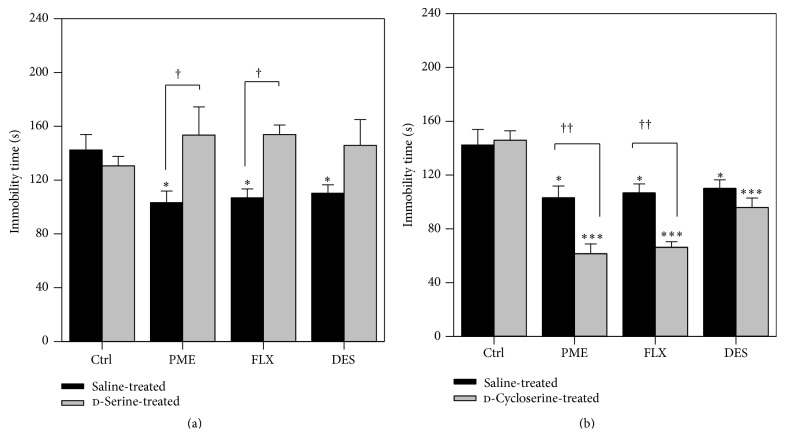
Effect of joint administration of d-serine (DS) or d-cycloserine (DCS) and PME, fluoxetine (FLX), or desipramine (DES) on the total duration of immobility in the TST in mice. The values represent means ± SEM of 5 mice. ^*∗*^ *P* < 0.05; ^*∗∗∗*^ *P* < 0.001 versus vehicle-treated animals (one-way ANOVA followed by Newman-Keuls' test). Significant difference between treatments: ^†^ *P* < 0.05; ^††^ *P* < 0.01 (two-way ANOVA followed by Bonferroni's test).

The effects of a combined administration of DCS and PME, FLX, or DES on total duration of immobility in mice are shown in Figure 7(b). Administration of DCS at a dose of 2.5 mg kg^−1^ had no effect on the immobility time in mice. Concomitant administration of DCS (2.5 mg kg^−1^) with PME (100 mg kg^−1^) significantly reduced the immobility time in mice (*F*_1,24_ = 16.42, *P* = 0.0037) with Bonferroni's* post hoc* analysis showing significance of *P* < 0.01. Similar effects were observed for fluoxetine (*P* < 0.01) but not desipramine.

The effects of a combined administration of PME, FLX, or DES and d-serine on total duration of immobility in mice are shown in Figure 7(a). PME (100 mg kg^−1^), FLX (10 mg kg^−1^), and DES (10 mg kg^−1^) significantly reduced the immobility time in mice as revealed by ANOVA (*F*_3,16_ = 4.483, *P* = 0.0182). d-Serine given alone at a dose of 600 mg kg^−1^ had no effect on immobility time but when combined with PME or FLX abolished their antidepressant-like effects (*F*_1,24_ = 8.849, *P* = 0.0177). d-Serine however did not completely abolish the anti-immobility of DES (*P* > 0.05).

#### 3.3.6. Involvement of L-Arginine-NO-cGMP Pathway

**Figure 8:**
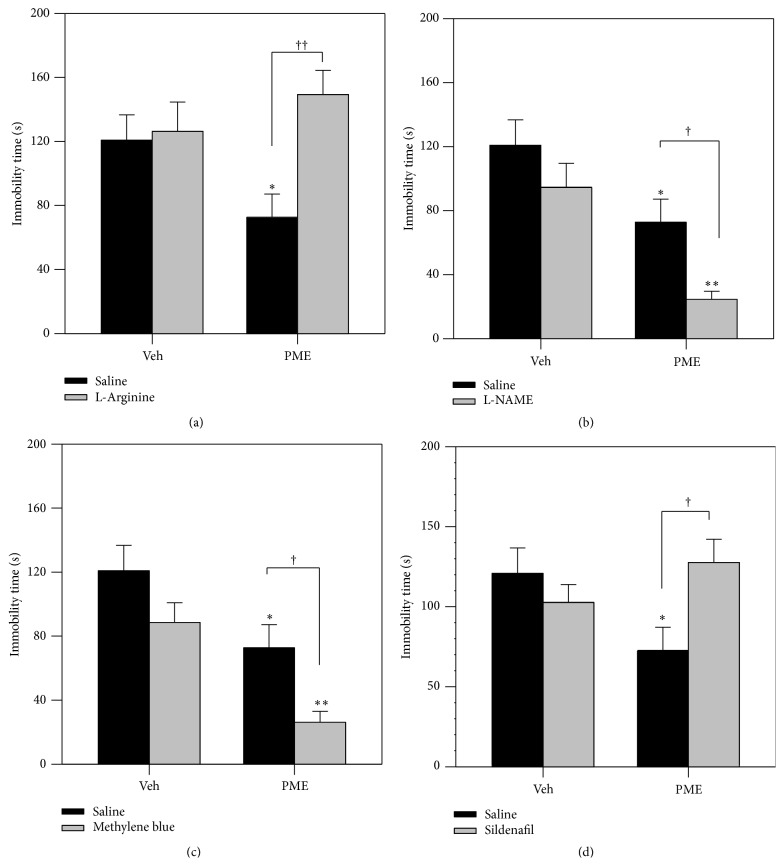
Effects of pretreatment of mice with L-arginine (750 mg kg^−1^, i.p., a precursor of nitric oxide, panel (a)), L-NAME (30 mg kg^−1^, i.p., a nonselective nitric oxide synthase inhibitor, panel (b)), methylene blue (10 mg kg^−1^, i.p., an inhibitor of NO synthase and an inhibitor of sGC, panel (c)), and sildenafil (5 mg kg^−1^, i.p., a phosphodiesterase 5 inhibitor, panel (d)) on PME- (100 mg kg^−1^,* p.o.*) induced reduction in immobility time in the TST. Each column represents the mean ± SEM (*n* = 6). ^*∗*^ *P* < 0.05; ^*∗∗*^ *P* < 0.01 versus vehicle-treated animals (one-way ANOVA followed by Newman-Keuls' test). Significant difference between treatments: ^†^ *P* < 0.05; ^††^ *P* < 0.01 (two-way ANOVA followed by Bonferroni's test).

Figure 8 shows the involvement of the nitric oxide pathway on the effects of PME in the TST. PME (100 mg kg^−1^,* p.o.*) significantly decreased the immobility time of mice in the TST (*P* < 0.05). Administration of L-arginine (750 mg kg^−1^, i.p., a precursor of nitric oxide) had no anti-immobility effects on mice in the TST compared with saline- (vehicle-) treated animals. However, pretreatment with L-arginine prevented the antidepressant-like effect of PME (100 mg kg^−1^,* p.o.*) with a Bonferroni* post hoc* analysis showing statistical significance of *P* < 0.01. L-NAME (30 mg kg^−1^, i.p., a nonselective nitric oxide synthase inhibitor) enhanced the antidepressant effect of an effective dose of PME (100 mg kg^−1^,* p.o.*) (*F*_1,20_ = 7.786, *P* = 0.0113). Methylene blue (10 mg kg^−1^, i.p., an inhibitor of NO synthase and an inhibitor of sGC) did not affect the immobility time in mice. However, methylene blue significantly enhanced the antidepressant effect of PME (*F*_1,20_ = 9.357, *P* = 0.0062).  Figure 8(d) shows that the pretreatment of animals with sildenafil (5 mg kg^−1^, i.p., a phosphodiesterase 5 inhibitor) significantly inhibited the reduction in immobility time elicited by PME in TST (*P* < 0.05).

### 3.4. Effect of PME on Rotarod Performance

**Figure 9:**
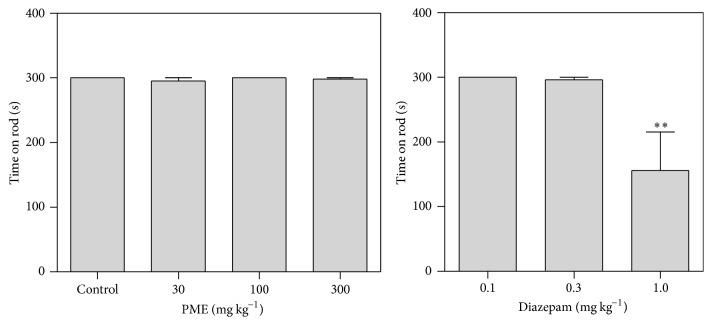
Behavioural effects of PME and DZP on muscle relaxant activity in the rotarod test in mice. Data are expressed as group mean ± SEM (*n* = 5). ^*∗∗*^ *P* < 0.01 compared to control group (one-way ANOVA followed by Newman-Keuls test).

Figure 9 shows the effect of PME on the performance of mice in the rotarod test. The extract caused no significant effect on the time taken by mice to fall off the rotarod compared to the control at all the doses used (*P* > 0.05 at 30–300 mg kg^−1^). Diazepam at the dose of 1.0 mg kg^−1^ caused significant decrease in the latency to fall off the rotating rod (*P* < 0.01).

## 4. Discussion

The present study provides convincing evidence that PME, when administered orally, produces an antidepressant-like effect in both the FST and TST and also elicits its action to a similar extent as the selective serotonin reuptake inhibitor fluoxetine.

The FST and TST are widely used for screening potential antidepressant agents and are sensitive and relatively specific to all major classes of antidepressants including tricyclic antidepressants (TCAs), selective serotonin reuptake inhibitors (SSRIs), monoamine oxidase inhibitors (MAOIs), and atypical antidepressants [16–18]. These tests are based on the observation that rodents, after initial escape-oriented movements, develop an immobile posture when placed in an inescapable stressful situation. If antidepressant treatments are given prior to the test, the subjects will actively persist in engaging in escape-directed behaviour for longer periods of time than after vehicle treatment [16, 22]. There is, indeed, a significant correlation between clinical potency and effectiveness of antidepressants in both models [32, 33].

In this study, the modified version of the FST, originally introduced by Detke et al. [17], was used, in which three specific types of behaviour, that is, immobility, climbing, and swimming, were measured. The modified FST distinguishes between passive responses (immobility) and active responses (increases in swimming or climbing) to stress [34]. Moreover, it widely measures the effects of SSRIs in mice [17]. Antidepressants acting through the serotoninergic system, including the SSRIs fluoxetine, sertraline, paroxetine, and citalopram, selectively increase swimming behaviour. In addition, the modified FST differentiates between antidepressants that work through serotoninergic mechanisms or noradrenergic mechanisms, as noradrenergic compounds selectively increased climbing behaviour [17, 35] and drugs with dual effects increased both swimming and climbing [36, 37]. In this study, PME in a similar fashion as fluoxetine induced a dose dependent reduction in the immobility time and an increase in the swimming behaviour, whereas no changes were observed in the climbing behaviour. This profile of action may suggest that the mechanism of the antidepressant-like activity of PME is related to the modulation of the serotoninergic system. In contrast to PME and fluoxetine, desipramine had no effect on swimming behaviour but instead increased climbing behaviour.

Modifications have been made in terms of the measurement of specific behavioural components of active behaviours in the TST to help differentiate between classical antidepressants and other compounds with antidepressant-like effects but with different mechanisms of actions, such as opiates [19, 20]. While traditional antidepressants that inhibit serotonin and/or noradrenaline reuptake decrease immobility and increase swinging behaviour, opioids, having decreased immobility, increase curling behaviour [20]. It was observed that administration of PME, fluoxetine, and desipramine showed significant antidepressant-like effects by decreasing immobility. PME did not significantly affect pedalling but caused an increase in time spent swinging and curling. Thus, PME in addition to its antidepressant-like activities could possibly have opioidergic properties. The TST has many advantages over the FST including the lack of hypothermic effects of cold water, the ability to test strains that may have motor deficits that make swimming difficult, and increased sensitivity to a wider range of antidepressant compounds [21, 37]. For this reason, the TST was used to assess the mechanisms by which PME elicits its antidepressant-like activity.

In FST and TST, false-positive results can be obtained with certain drugs, in particular, psychomotor stimulants, which decrease immobility time by stimulating locomotor activity [30, 31]. Anti-immobility effect of PME seems not to be associated with any motor deficits, since mice treated with PME did not impair motor coordination in the rotarod test. This indicates that the reduction of immobility time elicited by PME treatment in the TST and FST is unlikely due to a psychomotor-stimulant effect but rather an antidepressant-like effect of PME.

Several reports have suggested the involvement of serotoninergic and noradrenergic receptors in the mechanism of action of several classes of antidepressant drugs, including TCAs, SSRIs, and MAOIs, as well as in the pathophysiology of depression. The monoamine hypothesis of depression proposes that there is depletion in the levels of serotonin and noradrenaline in the central nervous system [38, 39] Therefore, the current effective treatments for depression are considered to elevate brain serotonin and/or noradrenaline neurotransmission [40, 41]. Thus, in this study, the possible involvement of these systems in the antidepressant-like effect of PME administered orally was investigated.

To confirm a possible contribution of the serotonin transmission in the antidepressant effect of PME, mice were pretreated with pCPA, an inhibitor of serotonin synthesis [23, 42]. Even though the depletion of 5-HT does not always produce behavioural depression, depletion of 5-HT with pCPA blocks the effects of fluoxetine in the FST and TST, while the effects of desipramine, which acts primarily as a norepinephrine reuptake inhibitor, are unaffected by 5-HT depletion [23, 35]. According to previous reports, pCPA at the present dose administered for three consecutive days was able to deplete the endogenous store of serotonin successfully without affecting the noradrenergic or dopaminergic levels [1, 23]. In this study, the antidepressant-like effect of PME was abolished by pCPA administration, suggesting that 5-HT in the brain is essential for its action in the TST. This effect demonstrates that the serotoninergic mechanism underlies the acute behavioural effects of PME on tests of depressive behaviour.

Administration of large doses of 5-HTP, a precursor of 5-HT, induces head twitches that occur spontaneously and irregularly, probably through a central action of 5-HT. Head-twitch response (HTR), induced by 5-HTP in mice, provides a simple method of determining specific activities of potentiators and antagonists for 5-HT in the central nervous system [43]. Administration of PME and fluoxetine potentiated 5-HTP-induced HTR in mice. This potentiation of HTR may be due to the PME or fluoxetine-mediated inhibition of the 5-HT reuptake and resulting increase of the content of 5-HT in synapses. This finding is consistent with the fact that pCPA pretreatment attenuated the anti-immobility activity of PME and fluoxetine in the TST. In contrast, desipramine significantly decreased the number of 5-HTP-induced head-twitch responses.

A role for 5-HT_2_ receptors in the action of some antidepressants has been shown. Preclinical data has shown that 5-HT_2A/2C_ antagonism has a significant role in the mechanism underlying the antidepressant-like effect of several antidepressants [42, 44]. Furthermore, a significant hypersensitivity of 5-HT_2_ receptors in the brain of depressed suicide victims has been observed in addition to elevated densities of 5-HT_2A_ receptors in depressed patients which showed significant reduction with a clinical recovery [1, 45]. In the present study, the anti-immobility effect elicited by PME in the TST was blocked by the pretreatment of mice with cyproheptadine (a 5-HT_2_ receptor antagonist), whereas prazosin, propranolol, and yohimbine had no effects. This further confirms the role of the 5-HT system in the mechanism of the antidepressant-like activity of PME.

The norepinephrine (NE) potentiation toxicity in mice reveals an adrenergic component of the pharmacological activity of antidepressants. Results of the present study showed that PME did not potentiate NE toxicity indicating noninvolvement of the noradrenergic system in its antidepressant-like effects. This further confirms the previous report in which the anti-immobility effect of PME was not reversed by prazosin (selective *α*_1_-receptor antagonist) and yohimbine (*α*_2_-receptor antagonist).

Several studies have implicated the NMDA class of glutamate receptors in the pathophysiology of depression and the mechanism of action of antidepressant treatment [27, 46, 47]. In addition, studies have reported antidepressant-like effects of a variety of NMDA receptor antagonists in animal models of depression such as the mouse FST [27, 48] and TST [49]. Thus, this study further assessed the involvement of NMDA receptors in the antidepressant-like effect of PME. d-Cycloserine is a partial agonist of glycine_B_ site of the NMDA receptor complex [10, 50]. At low doses, d-cycloserine (DCS) exerts an agonist profile as it mimics the action of endogenous glycine at its site and at higher doses competitively antagonizes the glycine site [10, 51]. In this study, DCS did not change the behaviour of animals in the TST; however, a potentiating effect was seen when DCS was given jointly with PME or fluoxetine. This apparent potentiation was manifested as a reduction of immobility time, which suggests a participation of the glycine site of the NMDA receptor complex in the antidepressant-like activity of PME. In another study, the influence of d-serine (a full agonist on glycine/NMDA receptors) on the activity of PME in the TST was evaluated. d-Serine did not change the immobility time in the TST; however, concomitant administration with PME, fluoxetine, and desipramine blocked their anti-immobility actions. It has been reported that d-serine blocked the antidepressant effects of imipramine, fluoxetine, and reboxetine, suggesting that activation of the glycine/NMDA receptor complex abolishes the antidepressant effects of both serotonin and noradrenaline-based compounds [10, 27, 52]. Interaction between NMDA receptor and serotoninergic pathway is more obvious than NMDA receptor and noradrenergic one. This is evident by the fact that NMDA receptor antagonists release/increase the concentration of serotonin and increase its turnover in the brain [53, 54]. Thus, it can be suggested that the antidepressant-like activity of PME (which acts via serotoninergic pathway) may occur* via* an effect on the glycine site of NMDA receptor.

Nitric oxide (NO) is a signalling molecule in the brain and has been implicated in neurotransmission, synaptic plasticity, learning, perception of pain, aggression, anxiety, and depression [55–57]. It has been reported that suicidal patients showed significantly higher levels of plasma nitric oxide metabolites than in nonsuicidal psychiatric patients or in normal control subjects [58, 59]. Similarly, depressed patients showed significantly higher plasma nitrate concentrations, suggesting that NO production is increased in depression [51, 60]. An appreciable number of studies have attributed a significant role to the L-arginine-NO-cGMP pathway in the pathophysiology of depression. Therefore, the possible participation of this pathway in the antidepressant effect of PME was investigated.

Results of the present study showed that pretreatment of mice with L-arginine, a NOS substrate, significantly inhibited the anti-immobility effect of the extract. Furthermore, a synergistic antidepressant-like effect was observed when PME was administered with N^G^ nitro-L-arginine-methyl ester (L-NAME), a nonselective nitric oxide synthase inhibitor, or methylene blue, an inhibitor of both NOS and sGC. Several studies have demonstrated that NOS inhibitors exert antidepressant-like effects in animal models predictive of antidepressant activity [61, 62]. Thus, these results indicate that the inhibition of NO synthesis may be involved in the antidepressant-like effect of PME in the TST. Several reports have shown that NOS inhibitors augment the behavioural effect of tricyclic antidepressants and selective serotonin reuptake inhibitors, but not noradrenaline reuptake inhibitors in the FST [51, 63, 64]. Therefore, the administration of NOS inhibitors could cause an enhanced effect of SSRIs in antidepressant therapy. It has been shown that PME elicits its anti-immobility effect via the 5-HT pathway devoid of the noradrenergic system. Therefore, the possible involvement of the nitric oxide system in the antidepressant-like effect of PME could be attributed to its interaction with the 5-HT system.

Heiberg et al. [61] demonstrated that excessive cGMP levels may produce a depression-like state and reducing its levels may produce antidepressant-like actions. The levels of cGMP can be decreased by either inhibiting the soluble guanylate cyclase (by methylene blue) or by decreasing the function of nitric oxide (by inhibiting NOS enzyme). In addition, cGMP is degraded into guanosine monophosphate (GMP) with the help of phosphodiesterase enzyme (PDE). Thus, inhibiting phosphodiesterase enzyme by using its inhibitors may increase the levels of cGMP and therefore produce a depression-like state [59]. Sildenafil is a selective PDE5 inhibitor that increases the cGMP level in target tissues [56, 65]. In the present study, the antidepressant-like effect of PME was reversed by pretreatment with sildenafil (a selective PDE5 inhibitor), indicating that the extract probably exerts its effect in the TST by decreasing cGMP levels. This is consistent with several reports in which sildenafil blocked the anti-immobility effects of compounds with antidepressant activity [66–68].

## 5. Conclusion

Results indicate that* Pseudospondias* extract produces an antidepressant-like effect in the FST and TST that is dependent on the serotoninergic system, NMDA receptor complex, and the L-arginine-NO-cGMP pathway. This suggests that* Pseudospondias microcarpa* could be of potential interest as a putative alternative therapeutic tool that could help the conventional pharmacotherapy of depression.

## Figures and Tables

**Table 1:** Effect of PME, fluoxetine, and desipramine on NE induced toxicity in mice.

Group	Dose (mg kg^−1^)	Number of deaths	% mortality
Control		0	0

PME	30	0	0
	100	0	0
	300	0	0

FLX	30	0	0

DES	30	5	50

Data indicates the number and percentage of mice (*n* = 10) that died.
